# Control of citrate utilization by *Candida albicans* Adr1

**DOI:** 10.1128/msphere.00311-25

**Published:** 2025-06-11

**Authors:** Amelia M. White, Aaron P. Mitchell

**Affiliations:** 1 Department of Microbiology, University of Georgia 1355, Athens, Georgia, USA; University of Michigan Michigan Medicine, Ann Arbor, Michigan, USA

**Keywords:** *Candida albicans*, carbon metabolism, transcriptional regulation, regulatory rewiring

## Abstract

*Candida albicans*, a fungal commensal and pathogen, occupies diverse niches in the human host. Its broad metabolic repertoire is critical for its survival. The model yeast *Saccharomyces cerevisiae* provides a starting point for analysis of *C. albicans* physiology and regulatory circuitry, but there are many examples of rewired transcription factors that govern different processes in the two organisms. We focus here on Adr1, which in *S. cerevisiae* promotes alternative carbon source utilization and in *C. albicans* promotes ergosterol synthesis. We find that *C. albicans* Adr1 is also required for growth on citrate and compounds that feed into the citric acid cycle, like glutamate and malate. RNA-sequencing (RNA-seq) shows that predicted citrate metabolic genes, representing both the citric acid cycle and gluconeogenesis, are downregulated in an *adr1*Δ/Δ mutant. In fact, the three Adr1-dependent genes *HGT17, MDH1,* and *PCK1* are required for growth on citrate, as indicated by deletion mutant phenotypes. The hyphal regulator *EED1* has a negative role in citrate utilization, and an *adr1*Δ/Δ *eed1*Δ/Δ double mutant is defective for growth on citrate. This result argues that Adr1 acts downstream or independently of Eed1 to govern citrate utilization. *C. albicans* Adr1 is rewired compared to its *S. cerevisiae* ortholog to govern the ability to use citrate, which *S. cerevisiae* lacks, and potentially to respond to Eed1, for which *S. cerevisiae* lacks an ortholog.

**IMPORTANCE:**

*Candida albicans* is a major fungal pathogen of humans, and its ability to grow on a range of carbon sources is critical for pathogenicity. Here, we find that a known regulator of ergosterol synthesis, Adr1, is also required to use citrate as a carbon source. Adr1 acts downstream or independently of Eed1, a well-known regulator of hypha formation and citrate utilization.

## INTRODUCTION

The fungus *Candida albicans* exists as both a commensal and a pathogen (1, 2). It survives in diverse host niches that include skin, mucosae, blood, and deep tissue (1, 3, 4). *C. albicans*’ ability to assimilate a range of carbon sources is essential for both its commensal and pathogenic states (1, 2, 5, 6). In fact, carbon metabolism is considered a useful source of potential antifungal drug targets (7).

*C. albicans* prefers glucose as a carbon source, and it maintains an elaborate signaling network to promote consumption of glucose when it is available (8, 9). Carbon control in the model yeast *Saccharomyces cerevisiae* has served as an excellent starting point to define key elements of carbon control in *C. albicans*. Some regulators, such as the Snf1-Mig1/Mig2 signaling module for control of alternative carbon utilization genes, are well conserved between the two species (9). Other regulators, such as the Gal4 transcription factor, are rewired to control different targets in *S. cerevisiae* and *C. albicans* (10–13). The distinctive regulatory features of *C. albicans* enable its adaptation to diverse host environments and nutritional scenarios (14, 15).

Our study focuses on the rewired transcription factor Adr1 (Alcohol Dehydrogenase II synthesis Regulator). *S. cerevisiae* Adr1 was among the first C_2_H_2_ zinc-finger proteins characterized in detail (16). In this yeast, Adr1 is required for expression of the alcohol dehydrogenase gene *ADH2* and additional genes necessary for utilization of alternative carbon sources (6, 11). The function of *C. albicans* Adr1 is different; it is not required for growth on ethanol, acetate, or glycerol (11). Instead, *C. albicans* Adr1 is a positive regulator of ergosterol biosynthesis (17). This function was deduced from the finding that an activated Adr1 derivative conferred resistance to the ergosterol-targeting antifungals fluconazole, amphotericin B, and terbinafine (17). ChEC-seq analysis indicated that Adr1 binds to the *ERG5, ERG11,* and *ERG28* upstream regions, and a mutation in the Adr1-binding site at *ERG11* severely impairs *ERG11* expression (17). Therefore, in *C. albicans*, Adr1 functions as a direct activator of ergosterol biosynthesis genes.

Our study also addresses the utilization of citrate, glutamate, and malate. These carbon sources are metabolized through the citric acid cycle and cannot be used as external carbon sources by *S. cerevisiae* (18). The Human Metabolome Database (19) indicates that all three are detectable in specimens of blood, breast milk, feces, saliva, sweat, and urine. These specimens represent sites of *C. albicans* colonization and infection. Moreover, given that all cells contain these molecules, tissue damage during infection may increase the three compounds’ availability. It makes sense then that *C. albicans* may be able to metabolize the compounds. In addition, it stands to reason that control of their utilization may be a distinctive feature of *C. albicans* not entirely shared with *S. cerevisiae*.

## RESULTS

### Role of Adr1 in alternative carbon source utilization

Previous studies indicated that a *C. albicans adr1*Δ/Δ transcription factor mutant has no growth defect on several gluconeogenic carbon sources (11, 17). We considered the hypothesis that other transcription factors may have overlapping functions and compensate for the absence of Adr1. Two candidates, Try4 and Zms1, were prioritized because (i) they are the top hits in a *C. albicans* BLASTP search with an Adr1 query, with alignment mainly over the C_2_H_2_ DNA-binding domains, and (ii) like Adr1, their RNA levels are controlled by glucose repression (12). We constructed a triple *adr1*Δ/Δ *try4*Δ/Δ *zms1*Δ/Δ mutant to investigate this hypothesis. However, the triple mutant had no growth defect on acetate (not shown). We then considered the second hypothesis that Adr1, Try4, and Zms1 govern utilization of carbon sources not tested previously. Growth tests with Biolog carbon source microarray plates (20) indicated that the triple mutant was defective in utilizing citrate, glutamate, and malate. Follow-up assays below showed that an *adr1*Δ/Δ single mutation was sufficient to cause these defects.

**Fig 1:**
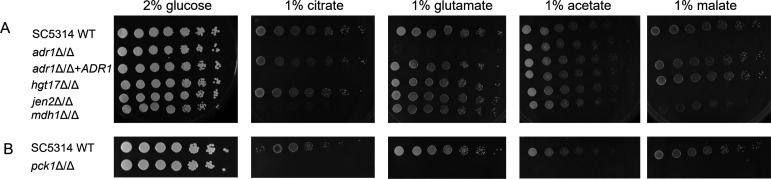
Growth properties of *adr1*Δ/Δ and Adr1 target gene mutants. Fivefold serial dilutions of the indicated strains were grown on YNB plus the indicated carbon sources for 48 h at 37°C. All strains are in the SC5314 genetic background. (**A**) Strains used were MC22 (WT), AW10 (*adr1*Δ/Δ), AW117 (*adr1*Δ/Δ + *ADR1*), AW157 (*hgt17*Δ/Δ), AW300 (*jen2*Δ/Δ), and AW66 (*mdh1*Δ/Δ). Overnights were grown in YNB + 1% acetate. (**B**) Strains used were MC22 (SC5314 WT) and AW75 (*pck1*Δ/Δ). Overnight cultures grown in YNB + 0.2% glucose because *pck1*Δ/Δ mutants cannot grow in acetate media.

We confirmed those preliminary findings with an independent *adr1*Δ/Δ mutant made in the SC5314 strain background. The *adr1*Δ/Δ mutant had a severe growth defect on citrate, glutamate, and malate, and no detectable defect on acetate or glucose in 37°C solid medium growth assays (Fig. 1A). A reconstituted strain with one *ADR1* allele inserted ectopically showed significant restoration of growth in citrate, glutamate, and malate (Fig. 1). Growth assays in liquid citrate medium, conducted at 30°C to minimize filamentation, confirmed defective growth of the mutant and improved growth of the reconstituted strain (Fig. S1A). The reconstituted strain grew slightly less well than the wild type, perhaps due to its reduced *ADR1* gene dosage. We conclude that Adr1 is required for growth on citrate, glutamate, and malate in the SC5314 strain background.

**Fig 2:**
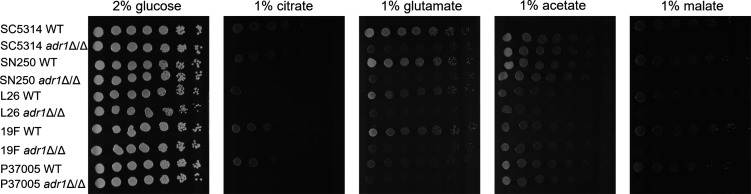
Growth properties of *adr1*Δ/Δ mutants in multiple strain backgrounds. Fivefold serial dilutions of the indicated strains were grown on plates containing YNB plus the indicated carbon sources for 48 h at 37°C. Strains used were MC22 (SC5314 WT), AW10 (SC5314 *adr1*Δ/Δ), MC310 (SN250 WT), AW51 (SN250 *adr1*Δ/Δ), MC46 (L26 WT), AW5 (L26 *adr1*Δ/Δ), MC48 (19F WT), AW11 (19F *adr1*Δ/Δ), MC31 (P37005 WT), and AW79 (P37005 *adr1*Δ/Δ).

*C. albicans* can infect diverse tissues that vary in carbon source availability (1, 5). We considered the possibility that strains isolated from different host niches may vary in dependence on Adr1. To explore this idea, we created *adr1*Δ/Δ mutations in multiple *C. albicans* backgrounds (21–23). We utilized strains isolated from the vulvovaginal region (L26 and 19F [23]) and the oral cavity (P37005 [23]). We compared them to SC5314 (the reference strain and bloodstream infection isolate obtained by a dermatologist [24]) and SN250 (an SC5314 derivative [25]). Although the wild-type clinical isolates differed in growth ability on citrate, glutamate, and malate, the *adr1*Δ/Δ mutants were uniformly defective (Fig. 2). In addition, we detected no growth difference between wild types and respective mutants on either glucose or acetate (Fig. 2). These results indicate that Adr1 is required for citrate, glutamate, and malate utilization in *C. albicans* isolates from a range of host niches.

### Adr1-dependent gene expression

**Fig 3:**
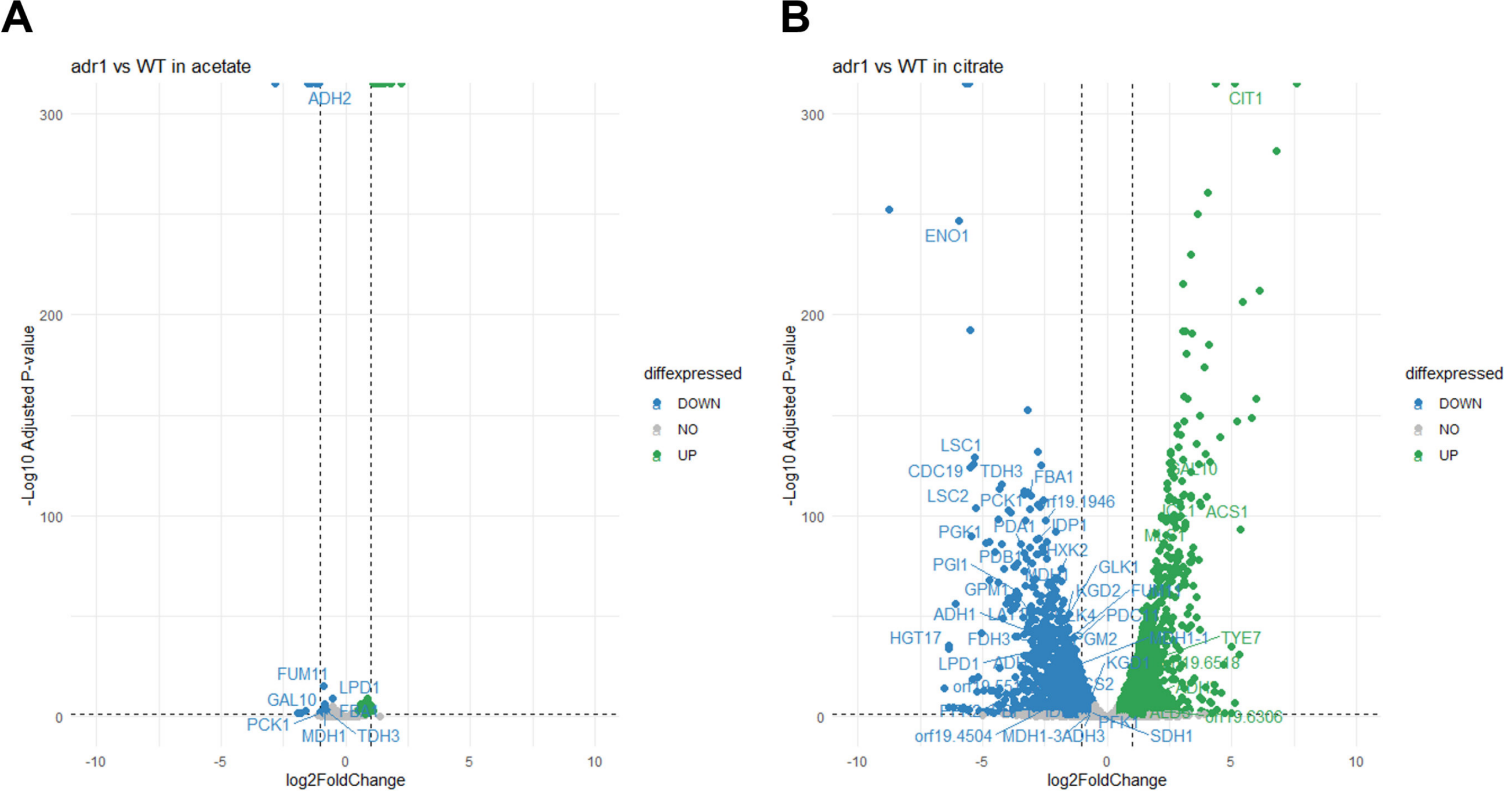
RNA-seq analysis. Images show volcano plots of differentially expressed genes; glycolytic genes and citric acid cycle genes are labeled. Blue dots represent significantly downregulated genes (log2fold change < −1, *P*adj < 0.05), green dots represent significantly upregulated genes (log2fold change > 1, *P*adj < 0.05). (**A**) Comparison of *adr1*Δ/Δ vs WT in YNB + 1% acetate media. (**B**). Comparison of *adr1*Δ/Δ vs WT in YNB + 1% citrate media. All strains are in the SC5314 genetic background; three biological replicates used for each strain and condition.

We used RNA-sequencing (RNA-seq) to define the gene expression impact of Adr1. Our initial analysis was conducted in acetate medium. The rationale was that a permissive carbon source like acetate might allow detection of Adr1-responsive genes without their being overshadowed by stress responses arising from a growth defect. In acetate medium, only 53 genes had altered RNA levels (18 downregulated, 35 upregulated) in the *adr1*Δ/Δ strain compared to the wild type, applying conventional thresholds of log_2_ fold change (LFC) <−1 or >1 and an adjusted *P*-value < 0.05 (Fig. 3A; Table S1). That gene set lacked significant GO term enrichment. With a relaxed LFC threshold (<−0.5 or >0.5), we identified 164 genes with altered RNA levels (53 downregulated, 111 upregulated [omitting *ADR1* and *HIS1* due to their deletion in strain construction]). This gene set included several carbon metabolic genes (e.g., *FBA1, FUM11, MDH1, PCK1,* and *TDH3*). These results indicate that, with acetate as a carbon source, Adr1 is required for full expression levels of several carbon metabolic genes, though the magnitude of its impact is quite modest.

The Adr1-dependent genes mildly affected during acetate growth might explain the failure of an *adr1*Δ/Δ mutant to grow on citrate, as discussed below. Therefore, we sought to assay Adr1-dependent gene expression by comparing the wild type and *adr1*Δ/Δ mutant in citrate medium. To determine a suitable sampling time, we conducted preliminary Nanostring assays with custom probes for 39 target RNAs and 4 normalization RNAs (Table S2). Cells were sampled after 3, 6, 9, and 12 hours of incubation in citrate medium. Many citrate metabolic genes were Adr1-dependent at all time points (see examples in Fig. S2). However, the magnitude of the *adr1*Δ/Δ mutant defect was greater in citrate than in acetate (Fig. S2; Table S2). Because expression defects were evident throughout the time course, we chose to examine a 4 hour time point in citrate medium, as we had in acetate medium, by RNA-seq.

RNA-seq data from citrate medium revealed that 1,995 genes had altered RNA levels (1,133 downregulated, 862 upregulated) in the *adr1*Δ/Δ strain compared to the wild type, again with thresholds of LFC <−1 or >1 and an adjusted *P*-value < 0.05 (Fig. 3B; Table S1). The Adr1-binding motif (17), 5′NRCCCCM3′, was present in 1,066 promoter regions of affected genes (Table S3). The upregulated set was enriched for diverse transcription factor genes (e.g., *EFG1, GLN3, RIM101, TAC1*, and *TYE7* [Table S3]). The downregulated set was enriched for diverse metabolic genes representing such processes as amino acid biosynthesis (e.g., arginine, isoleucine-valine, leucine, methionine), ergosterol biosynthesis, and the citric acid cycle (Table S3). These results indicate that, with citrate as a carbon source, Adr1 has a global impact on gene expression. The large impact of the *adr1*Δ/Δ mutation on gene expression may be a consequence in part of the mutant citrate growth defect, as we had anticipated.

**Fig 4:**
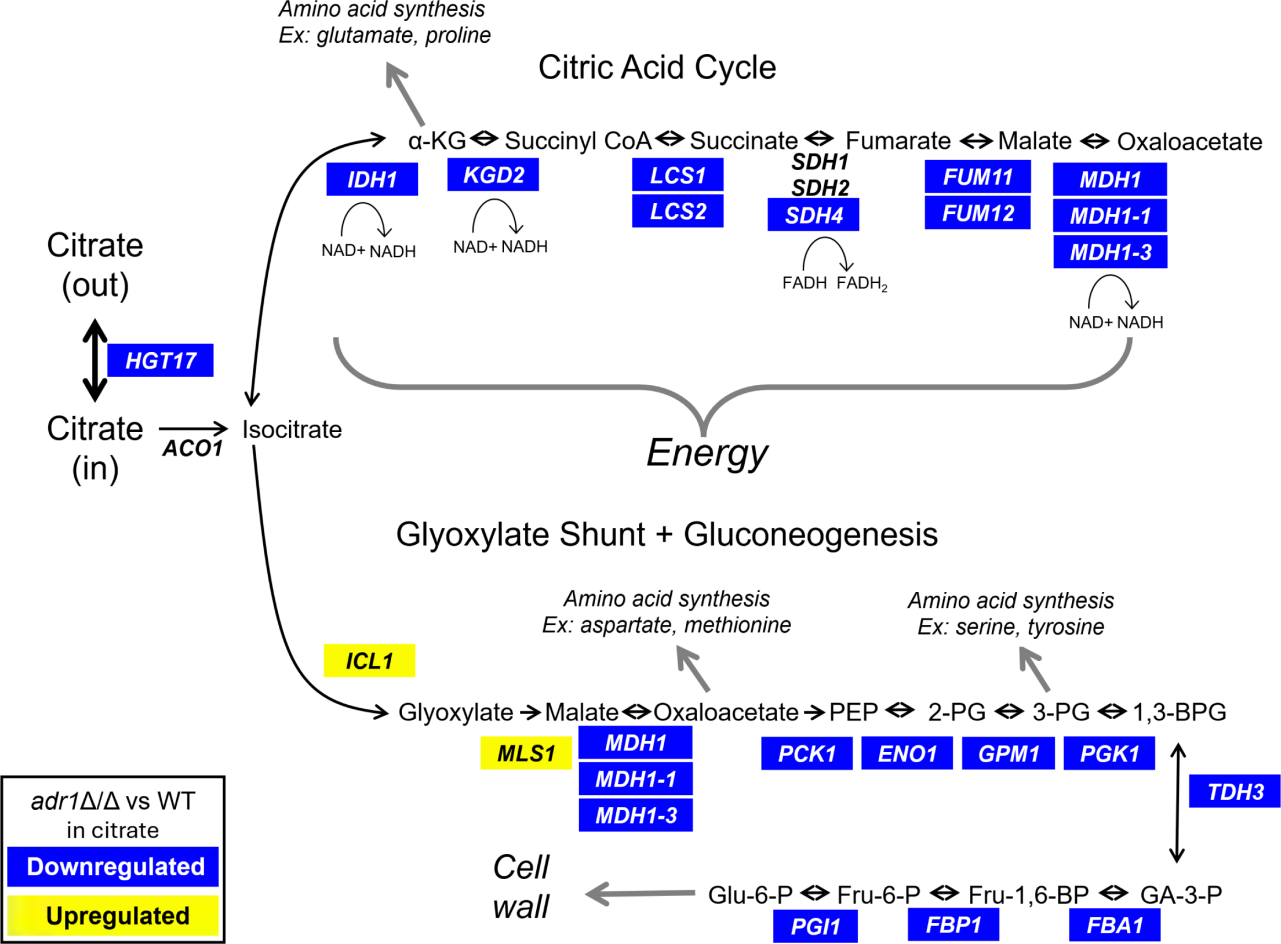
Adr1-dependent gene expression changes for predicted citrate metabolic genes. Predicted major citrate metabolic pathways for *C. albicans* are shown, inferred from annotations in the CGD (26) and KEGG (27) databases and our studies here of *HGT17*. We expect citrate to be metabolized through citric acid cycle enzymes to yield reducing equivalents for energy and to yield α-ketoglutarate for amino acid synthesis. We expect citrate to be metabolized through the glyoxylate shunt and gluconeogenesis to yield glucose-6-phosphate for cell wall biogenesis and to provide oxaloacetate and 3-phosphoglycerate for amino acid synthesis. For *adr1*Δ/Δ mutant cells compared to wild type cells in citrate medium, genes named in blue boxes are downregulated, genes named in yellow boxes are upregulated, and genes named without a box are not significantly altered.

To understand the basis for the *adr1*Δ/Δ growth defect, we assembled the major citrate metabolic pathways that are expected in *C. albicans*, based on annotations in the Candida Genome Database (CGD) (26) and the Kyoto Encyclopedia of Genes and Genomes (KEGG) (27), as well as information on *HGT17* below. Genes and enzyme activities are depicted in Fig. 4. We expect citrate to be metabolized through citric acid cycle enzymes to yield reducing equivalents for energy and to yield α-ketoglutarate for amino acid synthesis. We expect citrate to be metabolized through the glyoxylate shunt and gluconeogenesis to yield glucose-6-phosphate for cell wall biogenesis and to provide oxaloacetate and 3-phosphoglycerate for amino acid synthesis. Of the 28 genes that specify relevant gene products, 23 are expressed at reduced levels in the *adr1*Δ/Δ mutant compared to the wild type in citrate medium (Fig. 4). Therefore, the *adr1*Δ/Δ gene expression defect offers a simple explanation for the *adr1*Δ/Δ growth defect.

### Adr1 target gene function in growth

To validate Adr1 expression targets that may govern growth on citrate, we chose three genes that have not been extensively characterized in *C. albicans: MDH1*, *PCK1*, and *HGT17*. All three genes were substantially downregulated in the *adr1*Δ/Δ mutant on citrate (LFC < −2 for *adr1*Δ/Δ vs WT [Table S1]). In addition, they are likely to be direct Adr1 targets for two reasons. First, all three genes have the Adr1 binding motif (17), 5′NRCCCCM3′, in their 5′ regions. Second, all three are significantly downregulated in the *adr1*Δ/Δ mutant on acetate (LFC < −0.8 for *adr1*Δ/Δ vs WT [Table S1]), when the overall impact of Adr1 on gene expression is minimal.

*MDH1* (CR_00540C) specifies a predicted mitochondrial malate dehydrogenase, a citric acid cycle enzyme. *MDH1* is one of three paralogous genes, along with *MDH1-1* (C4_01900C) and *MDH1-3* (C2_10480W). The three gene products are ~50% identical and ~70% similar to one another. Although all three genes were downregulated in the *adr1*Δ/Δ mutant in citrate and acetate, *MDH1* was the most strongly downregulated (Table S1). We found that an *mdh1*Δ/Δ mutant, constructed in the SC5314 background, was severely growth defective on citrate, mildly growth defective on malate, and presented no defect on glutamate or acetate (Fig. 1A). The citrate growth defect was verified with assays in liquid medium (Fig. S1B). These results indicate that *MDH1,* like *ADR1,* is required for growth on citrate. The paralogs *MDH1-1* and *MDH1-3* may compensate for the loss of Mdh1 on glutamate, malate, and acetate media, though surprisingly not on citrate medium. Our results support the hypothesis that reduced *MDH1* expression contributes to the *adr1*Δ/Δ mutant growth defect on citrate medium.

*PCK1* (CR_00200W) specifies a predicted phosphoenolpyruvate carboxykinase, an enzyme critical for gluconeogenesis. We found that a *pck1*Δ/Δ mutant, constructed in the SC5314 background, was defective for growth on citrate, glutamate, malate, and acetate (Fig. 1B). The citrate growth defect was once again verified with assays in liquid medium (Fig. S1C). These results are consistent with the known role of phosphoenolpyruvate carboxykinase in gluconeogenesis. Our results support the hypothesis that reduced *PCK1* expression also contributes to the *adr1*Δ/Δ mutant growth defect on citrate medium.

*HGT17* (C4_01070W) specifies a putative glucose transporter that is a member of the major facilitator superfamily (28). Phylogenetic analysis places it in a distinct sugar transporter clade (“CaHgt”) that is divergent from *S. cerevisiae* family members (28). It is glucose-repressed and under the control of the Mig1/2 repressors (12). Moreover, *HGT17* is among the most highly upregulated genes in wild-type cells incubated in citrate, compared to acetate (Table S1). We found that a *hgt17*Δ/Δ mutant, constructed in the SC5314 background, was defective for growth on citrate but not on glutamate, malate, or acetate (Fig. 1A). The citrate growth defect was verified with assays in liquid medium (Fig. S1D). A control strain carrying a *jen2*Δ/Δ mutation, which affects a dicarboxylic acid transporter (29), was defective for growth on malate, but not on citrate, glutamate, or acetate, as expected (Fig. 1). Our results support the hypothesis that reduced *HGT17* expression also contributes to the *adr1*Δ/Δ mutant growth defect on citrate medium. In addition, our results are consistent with the idea that *HGT17* encodes a citrate transporter.

### Relationship between Adr1 and Eed1

**Fig 5:**
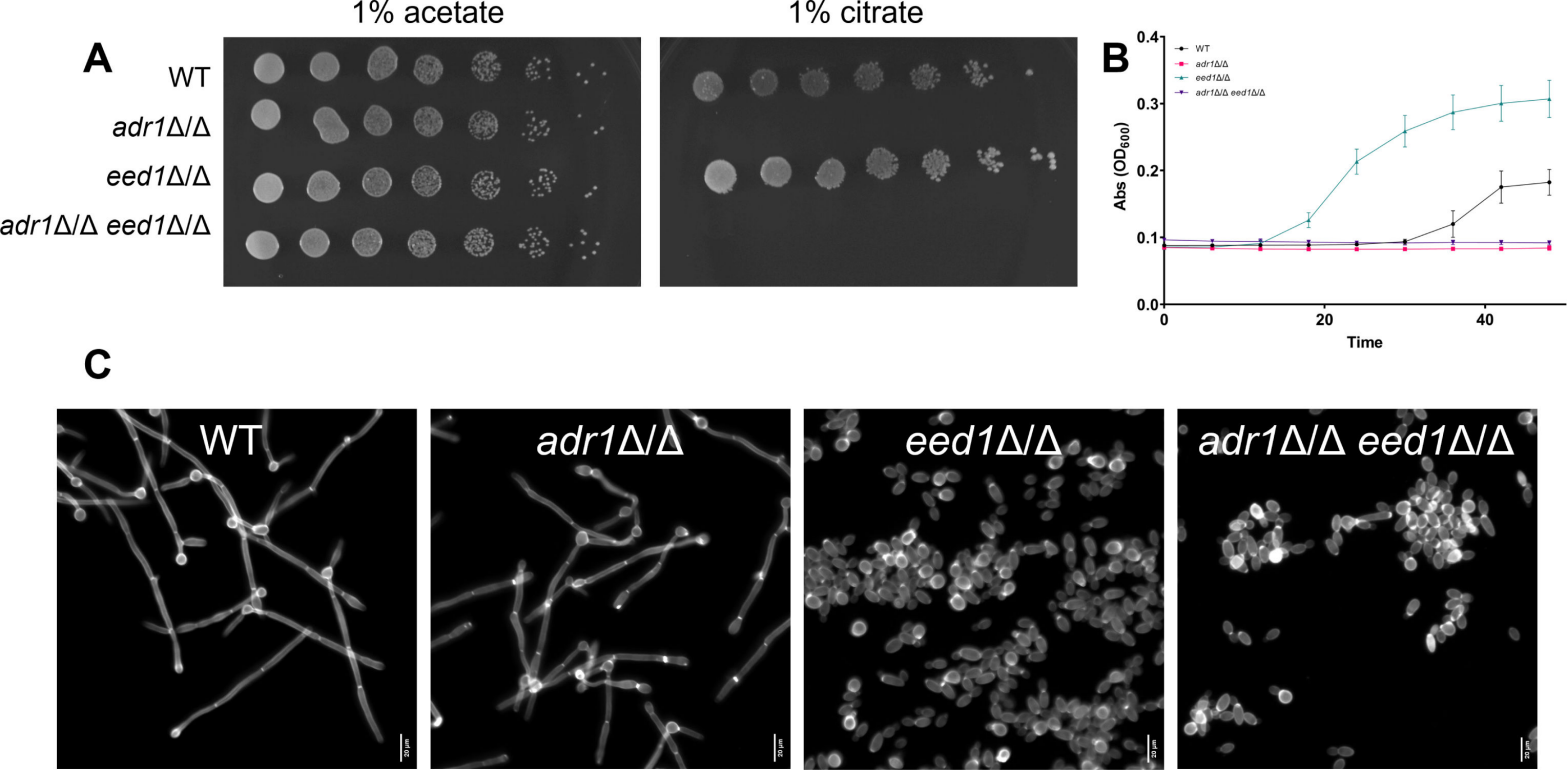
Genetic interaction of Eed1 and Adr1. Wild type, *adr1*Δ/Δ, *eed1*Δ/Δ, and *adr1*Δ/Δ *eed1*Δ/Δ strains in the SC5314 background were assayed for growth and morphogenesis. (**A**) Fivefold serial dilutions of each strain were grown on YNB plus the indicated carbon sources for 72 h at 37°C. All strains are in the SC5314 genetic background. Overnights were grown in YNB + 1% acetate. (**B**) Cultures were grown in 96-well plates at 30°C and inoculated from YNB + 1% acetate overnights. Three biological replicates were used for all samples. (**C**) Cell morphology was examined after 4 h at 37°C in RPMI medium. Cells were stained with calcofluor white and imaged by fluorescence microscopy. Scale bars represent 20 µm.

The *EED1* gene (epithelial escape and dissemination [30]); also designated CR_09880W and *DEF1* [RNAPII degradation factor] [26]) is well known as a positive regulator of filamentation. A recent study showed that *EED1* also has a negative role in adaptatng to citrate growth (31). Specifically, an *eed1*Δ/Δ mutant was found to adapt to growth on citrate more rapidly than the wild type, a phenotype we confirmed in solid and liquid media with an independently constructed *eed1*Δ/Δ mutant (Fig. 5A and B). To our knowledge, *ADR1* and *EED1* are the only two regulatory genes known at this time to govern the growth of *C. albicans* on citrate. (We note that the Zn(II)_2_Cys_6_ transcription factor ClrB has been shown recently to control citrate utilization in the basidiomycete yeast *Rhodotorula* [*Rhodosporidium*] *toruloides* [32].) To explore the functional relationship between them, we examined the growth properties of an *eed1*Δ/Δ *adr1*Δ/Δ double mutant. We found that the double mutant was severely defective in growth on citrate, based on assays in both solid and liquid media (Fig. 5A and B). In other words, the *adr1*Δ/Δ mutation is epistatic to the *eed1*Δ/Δ mutation for growth on citrate. We conclude that the impact of Eed1 on citrate utilization depends upon Adr1.

We also tested the double mutant for filamentation. The wild type and *adr1*Δ/Δ single mutant produced hyphae efficiently in RPMI medium; the *eed1*Δ/Δ single mutant did not, as expected (Fig. 5C). The *adr1*Δ/Δ *eed1*Δ/Δ double mutant also failed to produce hyphae. We conclude that the impact of Eed1 on hypha formation does not depend upon Adr1.

## DISCUSSION

We have shown that *C. albicans* transcription factor Adr1 is required for growth on citrate and compounds that feed into the citric acid cycle, including glutamate and malate. Adr1-regulated genes account for this requirement: Adr1 promotes expression of citric acid cycle and gluconeogenic genes, as well as the prospective citrate transporter gene *HGT17*. The hyphal regulator Eed1, which lacks a *S. cerevisiae* ortholog, also has a role in citrate utilization (31), and our data argue that Eed1 acts either upstream or independently of Adr1 to govern citrate utilization. Interestingly, Adr1 is rewired compared to *S. cerevisiae* in terms of its gene targets and, potentially, its regulatory relationship with Eed1.

Adr1 is required for full expression of numerous citrate metabolic genes in citrate medium, though the *adr1*Δ/Δ gene expression alteration in citrate medium is likely to include many indirect effects of carbon starvation. For that reason, we relied upon the *adr1*Δ/Δ gene expression alteration in acetate medium, where the mutant has no growth defect, to guide our selection of targets for analysis. Significantly reduced expression was observed for the genes prioritized for deletion analysis (*HGT17, PCK1,* and *MDH1*) and several other genes with predicted roles in citrate utilization (*FUM11, FBA1, PGK1,* and *TDH3*). Six of these genes (all except *PGK1*) also have predicted Adr1 binding sites (NRCCCCM [17]) in their 5′ regions (Table S3), and are thus plausible direct Adr1 target genes. These findings are consistent with a model in which Adr1 is required for growth on citrate because of its activation of *HGT17, PCK1, MDH1*, *FUM11, FBA1,* and *TDH3*.

*HGT17* is among the most highly upregulated genes in citrate vs. acetate (Table S1), as expected if it functions in citrate metabolism. The fact that Hgt17 lies in a phylogenetic subfamily not shared with *S. cerevisiae* (28), given that *S. cerevisiae* cannot use citrate as a carbon source (18), is also consistent with a specific connection between Hgt17 and citrate. We propose that Hgt17 is a citrate transporter. This idea is supported by two arguments. First, Hgt17 belongs to the major facilitator superfamily. Many family members in *C. albicans* are hexose transporters or provisionally annotated as such (26, 28). However, some family members are known to transport substrates other than sugars, including glycerol transporter Hgt10 (33) and inositol transporter Hgt15 (34). Therefore, the proposal that Hgt17 may transport a substrate other than a hexose has precedents. Second, it is required for *C. albicans* growth on citrate but not on other Adr1-dependent carbon sources such as glutamate and malate. The specificity of Hgt17 for citrate growth aligns with the proposed role in citrate transport.

Eed1 is best known as a positive regulator of hyphal extension and host cell damage (30, 31, 35). Surprisingly, though, Dunker et al. recently found that *eed1*Δ/Δ mutants are fully virulent in a mouse model of invasive candidiasis, despite their failure to grow as hyphae in infected tissue (31). They discovered that an *eed1*Δ/Δ mutant grows more rapidly than the wild type on numerous host-relevant carbon sources, including citrate. Therefore, Eed1 is a negative regulator of citrate utilization. Our double mutant analysis suggests that Eed1 functions either upstream or independently of Adr1 to control citrate utilization. However, the role of Eed1 in hyphal extension seems to be independent of Adr1, because both an *eed1*Δ/Δ single mutant and an *eed1*Δ/Δ *adr1*Δ/Δ double mutant are defective in hyphal growth. The sequence of Eed1 does not make a clear prediction about its mechanistic activity. Therefore, whether and how Eed1 may control Adr1 will require further study.

Adr1 was recently shown to function as a positive regulator of ergosterol biosynthesis in *C. albicans* (17). Why might a single transcription factor govern both the metabolism of citric acid cycle intermediates and ergosterol biosynthesis? One central connection between the two processes is the necessity for acetyl-CoA. Ergosterol biosynthesis begins with the condensation of two acetyl-CoA molecules. Utilization of citrate or glutamate requires the glyoxylate shunt, which depends upon acetyl-CoA for the synthesis of malate from glyoxylate. Utilization of malate requires citrate synthase, which depends upon acetyl-CoA, to yield α-ketoglutarate for amino acid synthesis. An interesting possibility is that acetyl-CoA levels may govern Adr1 activity.

## MATERIALS AND METHODS

### Media and culture conditions

Strains used in this study (Table S4) were maintained in 15% glycerol stocks and stored at −80°C. For use, strains were streaked on YPD agar plates (2% dextrose, 2% Bacto peptone, 1% yeast extract, and 2% Bacto Agar) and grown in overnight cultures for 18 h at 30°C with agitation. Overnights were usually grown in filter-sterilized YNB + 1% sodium acetate (VWR) liquid medium, though where necessary, they were grown in filter-sterilized YNB + 0.2% glucose or autoclaved YPD liquid medium (2% dextrose, 2% Bacto peptone, and 1% yeast extract), as specified in figure legends. Gene expression experiments were conducted with filter-sterilized YNB containing 1% sodium citrate (RPI) or 1% sodium acetate. Growth assays in liquid media used autoclaved YNB to which was added filter-sterilized 1% sodium citrate, 1% acetate, 1% sodium L-glutamate Monohydrate (AMBeed), or 1% sodium L-malate (MP biomedicals). Media were solidified for plate growth tests with 2% Bacto Agar. All *C. albicans* transformants were selected on CSM-HIS (0.67% yeast nitrogen base without amino acids, 0.079% CSM-HIS, and 2% dextrose) for His+ isolates, or CSM-LEU (0.67% yeast nitrogen base without amino acids, 0.079% CSM-LEU, and 2% dextrose) for Leu+ isolates, or CSM-ARG (0.67% yeast nitrogen base without amino acids, 0.079% CSM-ARG, and 2% dextrose) for Arg+ isolates. YPD + NAT (2% Bacto peptone, 2% dextrose, 1% yeast extract, and 400 mg/mL nourseothricin [clonNAT; Gold Biotechnology]) was used to select for nourseothricin-resistant isolates.

### Single-mutant strain construction

Manipulation of the *C. albicans* genome was conducted as previously described (36, 37). Transformation used the lithium acetate method (36, 38) or, as a backup, electroporation (39). All primers and plasmids employed in this work are listed in Table S3. Mutants were constructed using SN250, SC5314, or SN152 strain backgrounds. Marker cassettes were constructed using primers with 80 bp of homology to the gene of interest on either side of the ORF. The addition of the APM1_adapt_F sequence to the forward primer and the APM1_adapt_R sequence to the reverse primer was used to allow amplification of the auxotrophic marker of interest from plasmids pSN52 (*HIS1*), pSN40 (*LEU2*), pSN69 (*ARG4*), and pNAT (*NAT1*). To generate mutants, cells were transformed with 1 µg of CaCas9 DNA, 1 µg of an sgRNA gene targeted to the gene of interest, and 3 µg of the marker cassette PCR product as a repair template. Transformants were grown on CSM media which lacked the appropriate amino acids. Candidates were genotyped by PCR with YFG (Your favorite gene) check up /F and YFG check int /R to check for the absence of the ORF. And YFG check up /F and Marker check int /R (*HIS1*, *LEU2*, *ARG4*, *NAT1*) to check for the presence of the marker of interest.

### Double-mutant construction

Double mutants were constructed in a background that had been made sensitive to nourseothricin by recycling the *NAT1* marker at the *his1*Δ*/*Δ locus simultaneously with the deletion of *ADR1* using *HIS1*, following the procedure of Huang and Mitchell (40). The *EED1* locus was then deleted in the *adr1*Δ/Δ *NAT1*-sensitive strain using the *NAT1* marker. Marker cassettes were constructed using primers with 80 bp of homology to the gene of interest on either side of the ORF. The addition of the APM1_adapt_F sequence to the forward primer and the APM1_adapt_R sequence to the reverse primer was used to allow amplification of the marker of interest. *adr1*Δ/Δ (Nat sensitive) cells were transformed with 1 µg of CaCas9 DNA, 1 µg of an sgRNA targeted to the *EED1* gene, and 3 µg of the *NAT1* marker cassette PCR product as repair template. Transformants were plated on YPD + NAT (2% Bacto peptone, 2% dextrose, 1% yeast extract, and 400 mg/mL nourseothricin [clonNAT; Gold Biotechnology]) plates for 48 h. Candidates were genotyped by PCR with *EED1* check up /F and *EED1* check int /R to check for the absence of the ORF. And *EED1* check up /F and *NAT1* check int /R to check for the presence of the *NAT1* marker.

### Ectopic complementation

Reconstituted strains were constructed in a background that had been made sensitive to nourseothricin by recycling the *NAT1* marker at the *his1*Δ*/*Δ locus simultaneously with the deletion of *ADR1* using *HIS1*, following the procedure of Huang and Mitchell (40). *ADR1* reconstituted strains were constructed using an ectopic locus (*MDR1*), where the *MDR1* locus was replaced with an ADR1 promoter + ADR1 ORF + NAT1 marker cassette. This cassette was constructed by using primers MDR1_upstream_ADR1 5′ /F and ADR1 3′_NAT _5′ /R and amplified from SC5314 WT gDNA. The *NAT1* cassette was amplified from the pNAT plasmid using pNAT_adapt /F and pNAT 3′_MDR1_downstream /R. Mutants were generated by transforming 1 µg of CaCas9 DNA, 1 µg of an *MDR1* sgRNA ge°ne, and 1.5 µg of *MDR1-ADR1-NAT1* cassette. Transformants were plated on YPD + 400 µg/mL nourseothricin. Candidate transformants were then screened for integration of the cassette. Integration was verified using an *MDR1* check up /F primer with an *ADR1* check internal /R primer, as well as *MDR1* check up /F and a *NAT1* internal check /R.

### Carbon source microarray growth assays

Screening for carbon source utilization defects was done using Biolog carbon source microarray phenotype plates PM01 (catalog #12111) and PM02 (catalog #12112) following the manufacturer’s instructions. In short, strains were inoculated with carbon source-deficient inoculating medium and the fungal-specific color-changing dye. Strains were inoculated and incubated for 48 h at 30°C with no agitation, then qualitatively compared to the wild type strain by visual inspection to identify growth deficiencies.

### Agar plate growth assays

Strains were grown overnight in YNB + 1% acetate for 18 h at 30°C with agitation or in YNB + 0.2% glucose where indicated. Strains were washed twice with autoclaved ddH_2_O, then diluted to an OD_600_ of 3 in ddH_2_O. Fivefold serial dilutions were spotted on appropriate media using a multichannel pipette. Plates were incubated at 37°C for 72 h and imaged.

### Liquid growth assays

Strains were grown overnight in YNB + 1% acetate for 18 h at 30°C with agitation. Strains were then washed twice with ddH_2_O and diluted to an OD_600_ of 0.1 in the YNB + 1% citrate. Cultures were inoculated into 96-well flat-bottom plates (Fisher Brand Cat#FB012931) and grown at 30°C with continuous shaking in an Agilent BioTek 800 TS machine for 48 h to 72 h as indicated. OD_600_ readings were taken every 30 min. For some mutants, additional growth curves were conducted in 125 mL flasks with agitation (225 rpm). Incubation and preparation were as described for 96-well plates, except that strains were inoculated into 25 mL of media, the culture time was extended to 96 h, and the OD_600_ measurements were taken by a spectrophotometer.

### Filamentation assays in planktonic conditions

Filamentation assays were conducted as described previously by Huang et al. (22). Strains were grown overnight in liquid YPD for 18 h at 30°C with agitation. Strains were then inoculated to an OD_600_ of 0.5 into pre-warmed RPMI media. After inoculation, strains were grown at 37°C for 4 h with agitation. Filamentation samples were collected by centrifugation and fixed with 4% formaldehyde in PBS solution for 15 minutes by vortexing. Subsequently, the samples were washed twice with PBS and stained with Calcofluor-white. Cell imaging was conducted as previously described by Kim et al. (41).

### RNA extraction

For RNA sequencing, strains were inoculated in 25 mL YNB + 1% acetate and incubated at 30^◦^C with shaking (225 rpm) overnight. Cells were filtered through 0.45 µm Millipore filters and subsequently washed in 1 mL of the designated growth media. Prewarmed 125 mL flasks with 25 mL YNB + 1% of the needed carbon source were then inoculated to an OD_600_ of 0.2. Cultures were grown for 4 h at 37°C with agitation (225 rpm). Three biological replicates were used for sequencing. Cells were harvested via vacuum filtration and quickly frozen (−80°C) until RNA extraction. RNA extraction was done by disrupting cells with Zirconia beads (Ambion, Fisher Scientific, Waltham, MA) and isolating RNA using 25:24:1 phenol:chloroform:isoamyl alcohol in combination with a Qiagen RNeasy Mini Kit (Qiagen, Venlo, Netherlands), as previously described (42).

For Nanostring analyses, strains were inoculated in 25 mL YNB + 1% acetate and incubated at 30°C with shaking (225 rpm) overnight. Cells were filtered through 0.45 µm Millipore filters and subsequently washed in 1 mL of YNB + 1% citrate. Prewarmed 125 mL flasks with 25 mL YNB + 1% citrate were then inoculated to an OD_600_ of 0.2. Cultures were grown for 3 h, 6 h, 9 h, or 12 h at 37°C with agitation (225 rpm). A single determination was used for each time point. Cells were harvested at the designated time points via vacuum filtration and quickly frozen (−80°C) until RNA extraction. RNA extraction was done by disrupting cells with Zirconia beads (Ambion, Fisher Scientific, Waltham) and isolating RNA using 25:24:1 phenol:chloroform:isoamyl alcohol in combination with a Qiagen RNeasy Mini Kit (Qiagen, Venlo, Netherlands), as previously described (42).

### RNA-sequencing

RNA samples were shipped to Novogene for total mRNA sequencing and basic bioinformatic analyses. An amount of 1 µg RNA per sample was used as input material for RNA-seq. Sequencing libraries were generated by purifying messenger RNA from total RNA using poly-T oligo-attached magnetic beads. The first cDNA was synthesized using random hexamer primers, followed by sequencing of both ends of the cDNA fragments using the Illumina platform (Novogene). Sequencing reads were aligned to the *C. albicans* reference (Assembly 22) using Hisat2 v2.0.5. Differential expression analysis between two groups (three biological replicates per group) was performed using the DESeq2 v1.40.2 R package using alpha = 0.05 (43).

### Nanostring analyses

Nanostring analysis was performed as described in detail previously (39). Gene expression was measured using the nCounter SPRINT Profiler. For Nanostring analysis, 39 target genes and 4 normalization genes (CDC28, FKH2, GIN4, and ARP3) were chosen. For the assay, 15 ng of RNA was mixed with the Nanostring probe mix and was incubated at 65°C for 18 hours in a thermocycler. Samples were loaded onto the cartridge in accordance with the manufacturer’s instructions and placed into the nCounter SPRINT profiler for analysis and subsequent data collection.

### Data interpretation

Interpretations and hypotheses were always guided by the comprehensive information at the Candida Genome Database (26), FungiDB (44), and the KEGG database (27). GO term enrichments were determined with the GO Termfinder tool at the Candida Genome Database. Binding-site locations were determined with the PathoYeastract tools (45).

## Data Availability

Strains and plasmids are available upon request. The authors affirm that all data necessary for confirming the conclusions of the article are present within the article, figures, and tables. RNA-seq data have been deposited in the NCBI Gene Expression Omnibus with accession number GSE287330.

## SUPPLEMENTAL MATERIAL

Figure S1 msphere.00311-25-s0001.docx Quantitative growth curves in YNB + 1% citrate.

Figure S2 msphere.00311-25-s0002.docx Adr1-dependent RNA level changes during incubation in citrate medium.

Table S1 msphere.00311-25-s0003.xlsx RNA sequencing data sets.

Table S2 msphere.00311-25-s0004.xlsx Nanostring citrate time course data.

Table S3 msphere.00311-25-s0005.xlsx *adr1* vs wt citrate gene sets.

Table S4 msphere.00311-25-s0006.xlsx Strains, oligonucleotides, and plasmids.

ASM does not own the copyrights to Supplemental Material that may be linked to, or accessed through, an article. The authors have granted ASM a non-exclusive, world-wide license to publish the Supplemental Material files. Please contact the corresponding author directly for reuse.
