# Supplementary material for: Control of citrate utilization by Candida albicans Adr1
Source: mSphere. 2025 Jun 11;10(7):e00311-25. doi: 10.1128/msphere.00311-25 (PMC12306173; doi:10.1128/msphere.00311-25)

**Figure S1. Quantitative growth curves in YNB+1% citrate.** Three biological replicates were used for all samples. Cultures were grown in 96-well plates at 30°C, and inoculated from YNB+1% acetate overnights except as noted. (A) Comparison of growth of MC22 (WT), AW10 (adr1Δ/Δ), and AW117 (adr1Δ/Δ+ADR1). (B) Comparison of growth of MC22 (WT), AW10 (adr1Δ/Δ), and AW66 (mdh1Δ/Δ). These assays were conducted in 125mL flasks. (C) Comparison of growth of MC22 (WT), and AW75 (pck1Δ/Δ). Overnight cultures were grown in YNB+0.2% glucose as pck1Δ/Δ mutants cannot grow in acetate media. (D) Comparison of growth of MC22 (WT), AW10 (adr1Δ/Δ), and AW157 (hgt17Δ/Δ).


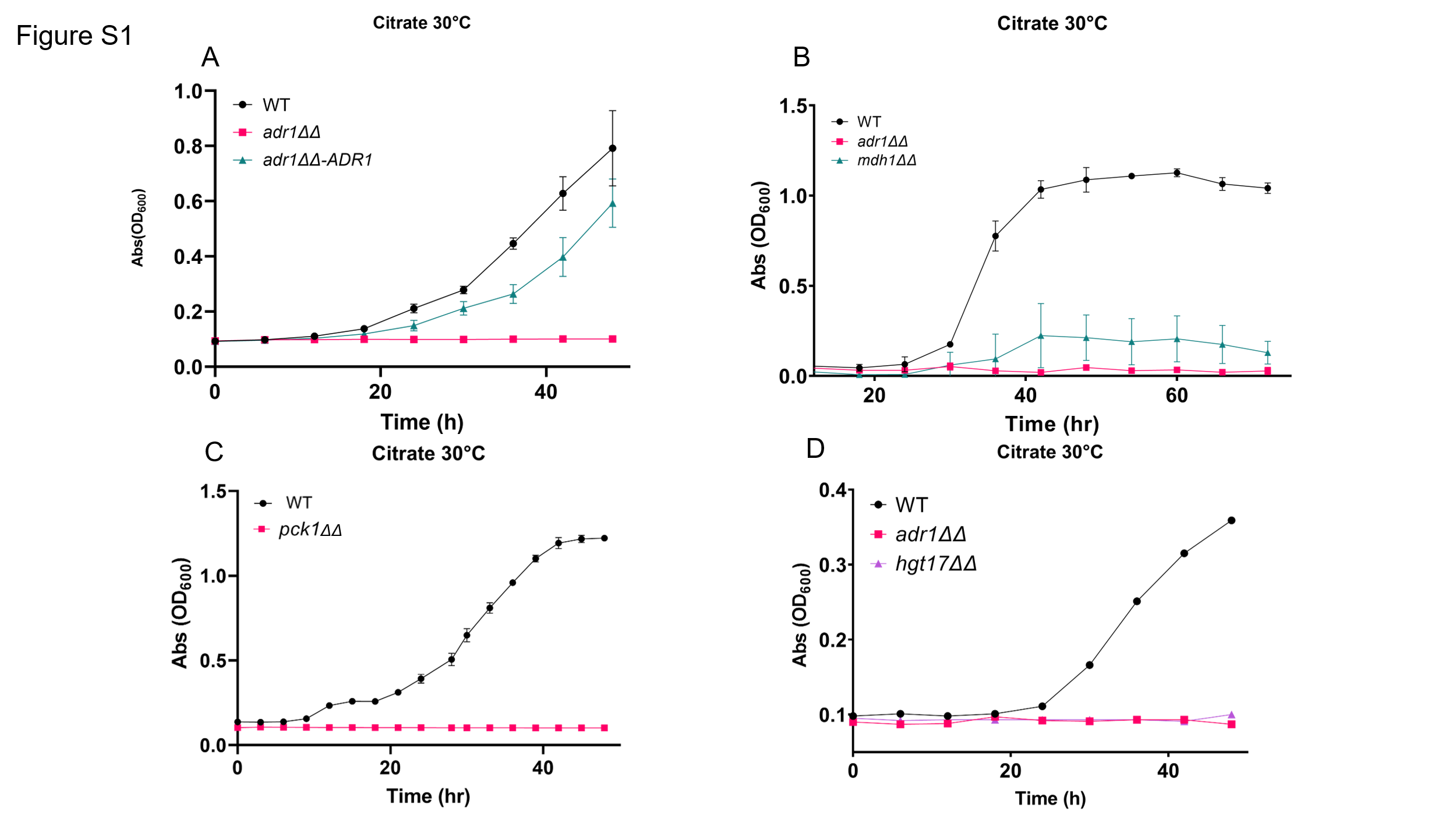

Supplement: Figure S1 — Quantitative growth curves in YNB + 1% citrate. [file msphere.00311-25-s0001.docx]
