# Supplementary material for: Control of citrate utilization by Candida albicans Adr1
Source: mSphere. 2025 Jun 11;10(7):e00311-25. doi: 10.1128/msphere.00311-25 (PMC12306173; doi:10.1128/msphere.00311-25)

**Figure S2. Adr1-dependent RNA level changes during incubation in citrate medium.** Results of single Nanostring determinations are shown. RNA samples were prepared from wild-type and *adr1*Δ/Δ strains after 3, 6, 9, and 12 hours of incubation in citrate medium at 37°C. RNA levels were assayed with a custom probe panel, normalized to four housekeeping genes, and log_2_ fold changes for the mutant were calculated. RNA samples from a 4 hour incubation in acetate medium were included for comparison. A few representative Adr1-dependent genes are shown; the complete dataset is in Table S2.


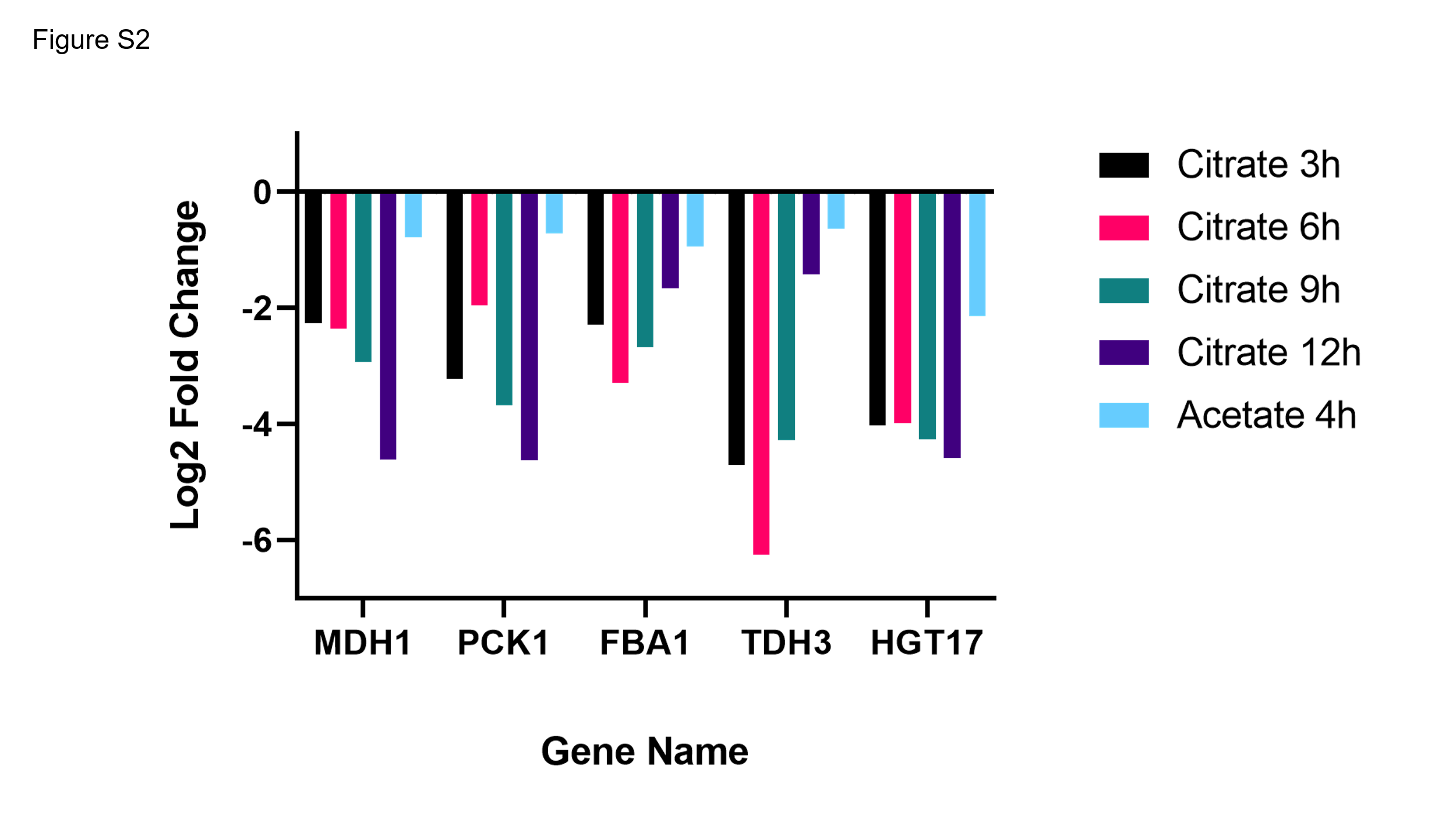

Supplement: Figure S2 — Adr1-dependent RNA level changes during incubation in citrate medium. [file msphere.00311-25-s0002.docx]
